# An Innovative Immunotoxin Design Against Allergy Based on the IL-33 Cytokine and the Ribotoxin α-Sarcin

**DOI:** 10.3390/ijms26199827

**Published:** 2025-10-09

**Authors:** Javier Narbona, Rodrigo Lázaro-Gorines, Adrián Gutiérrez-Carmona, Juan Carlos López-Rodríguez, Mayte Villalba, Javier Lacadena

**Affiliations:** 1 Department of Biochemistry and Molecular Biology, Faculty of Chemical Sciences, Complutense University, 28040 Madrid, Spain; jnarbona@ucm.es (J.N.); adgutier@ucm.es (A.G.-C.); mvillalb@ucm.es (M.V.); 2 Cancer Immunotherapy Unit, Immunology Department, Hospital Universitario 12 Octubre, Avda. de Córdoba, s/n, 28041 Madrid, Spain; rlazaro@cnio.es; 3 Immuno-Oncology & Immunotherapy Group, Instituto Investigación Sanitaria 12 de Octubre, Avda. de Córdoba, s/n, 28041 Madrid, Spain; 4 H12O-CNIO Cancer Immunotherapy Clinical Research Unit, Centro Nacional de Investigaciones Oncológicas (CNIO), 28029 Madrid, Spain; 5 Departamento de Ciencias Médicas Básicas, Facultad de Medicina, Instituto de Medicina Molecular Aplicada-Nemesio Díez (IMMA-ND), Universidad San Pablo-CEU, CEU Universities, Urbanización Montepríncipe, Ctra. Boadilla del Monte, Km. 5.300, 28668 Madrid, Spain; juan.lopezrodriguez@ceu.es

**Keywords:** immunotoxin, IL-33, ribotoxin α-sarcin, ILC2 cells, ST2 receptor, allergic response

## Abstract

Allergies constitute one of the major health problems worldwide, increasing their prevalence in developed countries. To overcome this multifactorial disease, immunotherapy and the use of immune molecules, such as immunotoxins, have arisen as promising therapeutic tools. We have designed, produced, and characterized a new immunotoxin called IL-33αS, encompassing the murine IL-33 (mIL-33) as the target domain and the ribotoxin α-sarcin as the toxic domain. IL-33 is a widely described alarmin that binds to the ST2 receptor of a variety of immune cells, including ILC2s, leading to Th2-derived inflammatory response, as occurs in allergic reactions. Both IL-33αS and mIL-33 were successfully produced in the methylotrophic yeast *Pichia pastoris* and purified to homogeneity through affinity chromatography for their characterization. Both IL-33αS and mIL-33 were able to specifically bind to ST2^+^ Raw 264.7 cells, and IL-33αS kept the ribonucleolytic activity of α-sarcin, allowing IL-33αS to exhibit cytotoxic effects against ST2^+^-targeted cells. In addition, IL-33αS induced significantly less secretion of the Th2-linked cytokine IL-13 in comparison to mIL-33, suggesting steric interference produced by the presence of the α-sarcin. These results assess the potential therapeutic effect of this new immunotoxin against allergies, causing ST2-targeted cytotoxicity while avoiding the Th2 cytokine secretion.

## 1. Introduction

Allergies and allergic-related pathologies have been estimated to affect up to 40% of the global population, implying a great burden on patient quality of life, healthcare systems, and the global economy [1], especially in developed countries over the recent years. Industrialization and changes in modern lifestyle have modified human exposure to different respiratory allergens, exacerbating the subsequent cases of allergic asthma and other Th2-derived pathologies [2,3], which are the clinical manifestations of the allergic disease asthma, rhinitis, conjunctivitis, and, in fatal cases, anaphylaxis [4].

During early stages of allergic inflammation, damage and homeostasis deregulation of the epithelial barrier causes abnormalities and dysfunctions of the immune basal state, which can lead to allergic processes, according to the epithelial barrier hypothesis proposed by Pothoven and Schleimer [5]. When endothelial and epithelial cells from this barrier are damaged, they secrete alarm signals, such as the alarmins IL-33, TSLP, and IL-25, that are able to activate downstream immune cascades. More precisely, IL-33 is a 30 kDa (270 aa) nuclear factor protein structurally related to the IL-1 and the Fibroblast Growth Factor (FGF) family [6]. The amino-terminal portion of IL-33 (aa 1–65) is responsible for compartmentalizing the cytokine into the nucleus and allows its binding to the chromatin. The central domain of IL-33 (aa 79–111 in humans) is a linkage domain that acts as a cleavage and recruitment platform for proteases, released during inflammation or infection [7]. Even the full-length protein is partially active, but when its central domain is cleaved by proteases from neutrophils (cathepsin G, elastase) [8] or from mast cells (such as chymase and tryptase proteases), this generates the fully mature forms, IL-33 (95–270), IL-33 (99–270), and IL-33 (109–270), which have 10 to 30 times higher biological activity [9].

Once secreted, IL-33, both the full-length and the mature form of the cleaved molecule, binds to its specific membrane receptor ST2 (growth stimulation expressed gene 2), which is present in a wide range of cells, specially tissue-resident immune innate cells, such as innate lymphoid cells type 2 (ILC2s), basophils, mast cells, and regulatory T cells among others. Once IL-33 is bound to ST2, the IL33-ST2 complex recruits the co-receptor IL1RAcP [10], leading to the formation of the heterodimer ST2-IL1RAcP, with the subsequent dimerization of the Toll-Interleukin-1-Receptor (TIR) domains of both receptors, and the downstream activation of the MAPK and NFκB pathways. Mainly, upon cell activation, ILC2s produce high amounts of Th2-linked cytokines such as IL-5 and IL-13; Th9-linked cytokines such as IL-9, IL-6, and IL-8; and CXCL10, ultimately leading to allergic inflammation, eosinophil recruitment and activation, and defense against helminth infection [11]. Current clinical guidelines and treatment include patient education, allergen avoidance, pharmacotherapy, and allergen-specific immunotherapy (AIT), with the latter being one of the best-established treatments for specific allergic conditions in Europe according to the European Academy of Allergy and Clinical Immunology (EAACI) [12]. Along with AIT, the use of antibodies and immunoconjugates, such as immunotoxins, against several diseases has arisen as promising therapeutic tools, within the idea of giving a more personalized medicine to the patient.

In this sense, immunotoxins are chimeric proteins that consist of a target domain, usually an antibody, an antibody fragment, or a cytokine that directs the action of a toxic domain, usually a toxin protein obtained from bacteria or plants, towards the target cell, leading to its death [13,14]. Regarding the toxic domain, fungal ribotoxins with RNase activity have arisen as promising candidates due to their high thermostability, resistance to proteases, low immunogenicity, and highly effective enzymatic activity [15], and especially the ribotoxin α-sarcin, the most studied and characterized member of this protein family [16]. Besides ribotoxins, other toxins such as RIPs (Ribosome-Inactivating Proteins), also included as toxic domains of different chimeras, have been the subject of numerous studies aimed at increasing their stability [17] or their pharmacological and biochemical properties [18], making them more adequate for treatment. Nowadays, immunotoxins have proven to be effective therapeutic tools against numerous kinds of cancer, both hematological [19] and solid tumors [20], for autoimmune diseases [21] or viral infections [22]. However, the use of immunotoxins to treat allergic processes has not been developed so much yet, even if some attempts have been made [23].

In this sense, we have designed, produced, and characterized a new immunotoxin against allergic inflammation, using as the target domain the murine cleaved active form of IL-33 (aa 109–270), and as toxic domain the ribotoxin α-sarcin, referring to this immunotoxin as IL-33αS. In addition, we have designed and produced just the murine cleaved mature active form of IL-33 (mIL-33) for its use as a control. As a first approach towards its use in humans, we have first designed these constructs against the mouse IL-33 receptor to validate their use against an allergic murine model, simpler than a human one. In this sense, we have carried out the characterization of these murine constructs to assess their relevance and allergic prevention in the more simplified mouse models.

## 2. Results

### 2.1. Production and Purification of mIL-33 and IL-33αS

**Figure 1:**
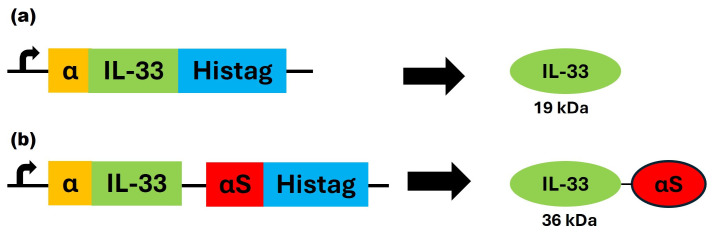
Schematic diagrams showing the gene and protein domains of mIL-33 (**a**) and the immunotoxin IL-33αS (**b**). On the left side, the DNA motifs are shown, whereas on the right side, the whole proteins are schematically represented, with their molecular size underneath. The domains with their different colors correspond to α-factor secretion signal sequence (α, yellow), the mature murine IL-33 cleaved sequence (aa: 109–270) (IL-33, green), the ribotoxin α-sarcin (αS, red), and the C-terminal histidine tag included to facilitate their purification (Histag, blue). A Gly-Gly-Arg (GGR) linker was included between mIL-33 and α-sarcin.

The plasmids containing the DNA sequences encoding for mIL-33 and IL-33αS were designed and purchased from IDT Technologies and cloned into the pPICZαA plasmid, obtaining the expression vectors pPICZαAmIL33 and pPICZαAIL33αS. These plasmids were electroporated into the KM71H *Pichia pastoris* strain (Figure 1).

**Figure 2:**
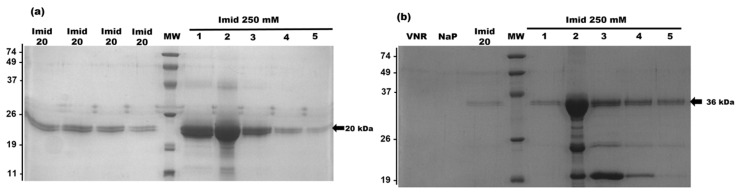
Coomassie blue-stained SDS-PAGE (15%) analysis of the purified fractions of the affinity chromatography of mIL-33 (**a**) and IL-33αS (**b**). Notes in gel correspond to the following: MW, molecular weight standard; NaP, washed fraction eluted with sodium phosphate buffer pH 7.5; Imid 20, washed fractions eluted with imidazole 20 mM sodium phosphate buffer; Imid 250 #1–5 and elution fractions with imidazole 250 mM sodium phosphate buffer. Molecular weight markers (MW) correspond, from top to bottom, to 74, 49, 37, 26, 19, and 11 kDa, respectively. Gels presented in the figure were slightly cropped. Original full-length gels are presented in Supplementary Figure S1.

Both proteins were successfully produced and secreted to the extracellular medium of the yeast. Purification was carried out as described in Section 4, and the presence of the proteins was confirmed with SDS-PAGE electrophoresis, followed by Coomassie blue staining (CBS) (Figure 2).

mIL-33 was present both in the 20 mM washing fractions and the elution fractions, with the expected molecular mass for the designed construct (20 kDa) (Figure 2a). As for the immunotoxin, IL-33αS appeared mainly in the elution fractions, with the expected molecular weight of 36 kDa, showing a mild degradation (Figure 2b).

**Figure 3:**
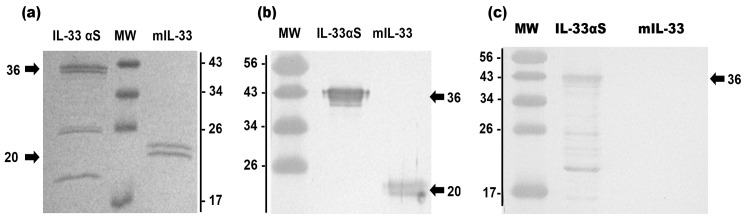
SDS-PAGE and Western blot analysis of IL-33αS and mIL-33: (**a**) Coomassie blue-stained SDS PAGE (15%) analysis of the final pool after the affinity chromatography purification step of mIL-33 and IL-33αS. Molecular weight marker (MW) values appear at the right. (**b**) Western blot analysis of both the purified mIL-33 and IL-33αS using an anti-IL33 monoclonal antibody. (**c**) Western blot analysis of both the purified mIL-33 and IL-33αS using an anti-α-sarcin antibody. All the molecular weights are shown in kDa. Images correspond to cropped gel and blot, respectively, acquired and analyzed using the Gel Doc XR Imaging System and Quantity One 1-D analysis software v4.6.9. (BioRad, Hercules, CA, USA) or ChemiDoc-It (UVP, Upland, CA, USA) and VisionWorks LS, v7.0 respectively. Original full-length gel and blot are presented in Supplementary Figure S2.

The elution fractions were collected and pooled for assessing the purity and identity of both proteins by SDS-PAGE, followed by CBS or Western blot using an anti-IL33 monoclonal antibody or an anti-α-sarcin antibody (Figure 3a–c, respectively). mIL-33 and IL-33αS were purified to homogeneity with a final yield of 15 and 5 mg/L of induction culture, respectively, with a slight degradation of IL33-αS.

### 2.2. Structural Characterization of IL-33αS

**Figure 4:**
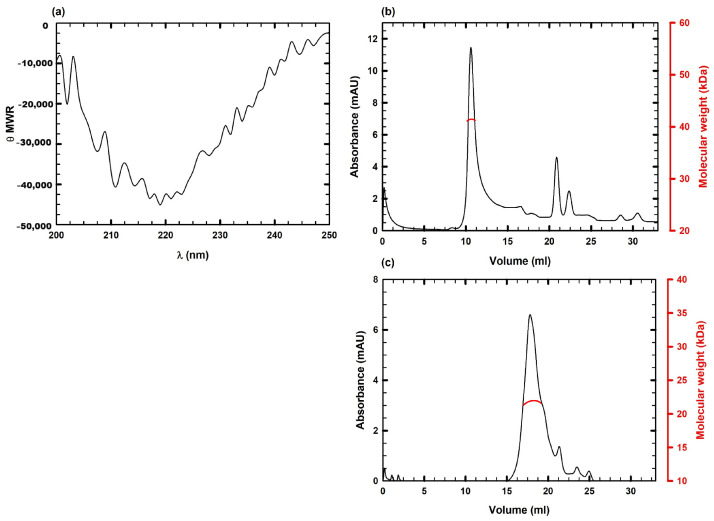
Structural characterization of IL-33αS: (**a**) Far-UV circular dichroism (CD) spectra of IL-33αS, at a final concentration of 0.2 mg/mL in 50 mM sodium phosphate, 0.1 M NaCl buffer, pH 7. θ_MRW_ represents the mean residue weight ellipticity as degree × cm^2^ × dmol^−1^. The HT signal obtained during Far-UV circular dichroism spectra measurements is shown in Figure S4. SEC analysis of the molecular weight in native conditions of IL-33αS (**b**) or mIL-33 (**c**) using a Superdex 200 column, showing the main peak molecular mass as red curves within the graphs, associated to the right axis in red.SDS-PAGE analysis of different fractions from IL-33αS SEC analysis is shown in Figure S5.

The structural characterization of the new immunotoxin IL-33αS was first analyzed through circular dichroism (CD) spectrometry, showing a final spectrum (Figure 4a) that was compatible with hydrosoluble proteins exhibiting a high content of β-sheet secondary structure. This structure was coherent with the main secondary structures of its domains, the IL-33 cytokine and the ribotoxin α-sarcin. An additional purification step using FPLC-size-exclusion chromatography (SEC) analysis was carried out after affinity chromatography shown in Figure 2, to confirm the monomeric state and molecular size of IL-33αS in native conditions. According to the results obtained, it was used as an additional purification step, leading to a final yield of 4 mg/L after this purification step. The elution profile obtained (Figure 4b) showed a main peak at the elution volume corresponding to 40 kDa, close to the expected for the molecular size of IL-33αS. For comparison, the elution profile for mIL-33 was also obtained, with a main peak corresponding to 20 kDa (Figure 4c). Secondary peaks of smaller molecular weight, corresponding to slight degradation, were observed in both cases. The degradation profile, before and after the SEC, was similar.

### 2.3. Ribonucleolytic Activity of Toxic Domain

**Figure 5:**
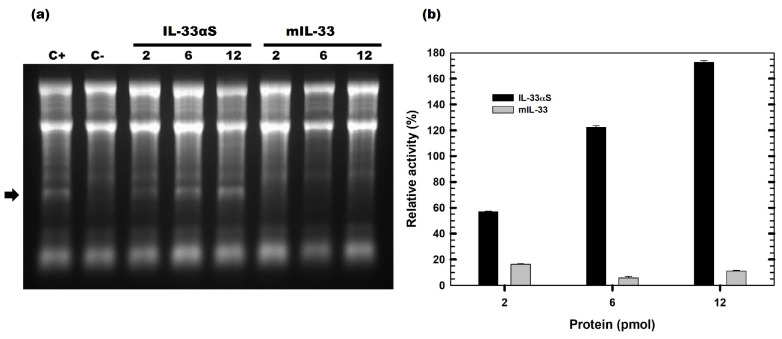
Functional characterization of the ribonucleolytic activity: (**a**) The agarose gel representing the ribonucleolytic activity of α-sarcin in IL-33αS and mIL-33. The arrow indicates the presence of the α-fragment. In both cases, 2, 6, and 12 pmoles of protein were tested, as indicated at the top of the gel. C+ represents 2 pmoles of fungal wild-type α-sarcin, and in C−, the protein sample was replaced by buffer. In total, 2, 6, and 12 pmoles of IL-33αS and mIL-33 were tested. The gel presented in the figure was slightly cropped. The full-length gel is presented in Supplementary Figure S3. (**b**) Quantitation of the specific ribonucleolytic activity of IL-33αS and mIL-33, expressed as the relative intensity of α-fragment/RNA 18 S ratio, considering 100% as the ratio obtained with 2 pmoles of wild-type α-sarcin. Triplicates of each condition were tested (n = 3).

Functional characterization of IL-33αS was first carried out by analyzing the proper function of both domains in the fusion protein IL-33αS. The toxic activity of the α-sarcin, located at the C-terminus of IL-33αS, was assayed by detecting the release of the characteristic 28S rRNA α-fragment produced by the specific ribonucleolytic activity of α-sarcin [15]. Figure 5a shows the presence of the α-fragment in all IL-33αS conditions, as a band whose intensity is higher as the amount of protein assayed is increased, reaching 12 pmoles of IL-33αS, up to 180% of the ribonucleolytic activity compared to the positive control, 2 pmoles of wild type α-sarcin (Figure 5b). On the contrary, as expected, mIL-33 did not lead to the liberation of the α-fragment, as it does not exhibit ribonucleolytic activity.

### 2.4. Binding Activity

**Figure 6:**
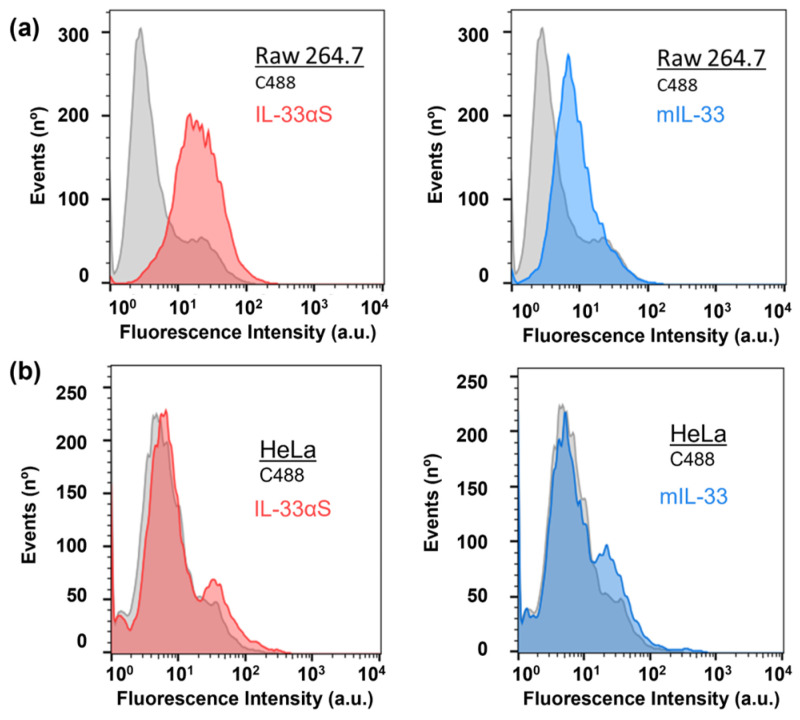
Binding activity of IL-33αS and mIL-33 against the Raw 264.7 cells (ST2^+^ cell line) (**a**) and against HeLa cells (ST2^−^ cell line) (**b**). Both IL-33αS (red) and mIL-33 (blue) were incubated with the cells, after conjugation with the fluorophore Alexa 488, at a final concentration of 1 µM of each protein, whereas the gray curves correspond to the cells incubated with an Alexa 488-conjugated anti-histidine antibody as an isotype control.

The specific targeting of mIL-33 and IL-33αS was assessed through flow cytometry experiments using the Raw 264.7 cell line as a murine ST2^+^ cell model [24,25]. As observed in Figure 6a, both IL-33-based proteins were able to specifically bind to the macrophage-like ST2^+^ cells, whereas no binding activity was observed to the ST2^−^ HeLa cell line (Figure 6b).

### 2.5. Cytotoxic Activity

**Figure 7:**
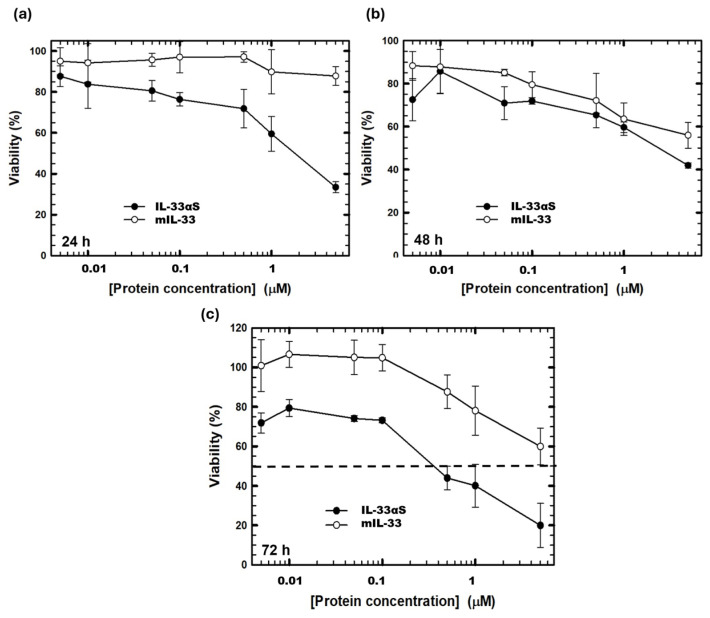
Cytotoxic characterization by MTT viability assays against Raw 264.7 cells, at 24 (**a**), 48 (**b**), and 72 h (**c**). Both IL-33αS (black) and murine IL-33 (white) were tested, and data was analyzed and plotted (mean ± SD) against untreated cell controls. In all the cases, triplicate tests of each condition were carried out. The horizontal dashed line represents 50% viability.

The combined activity of both targeting and toxic domains of IL-33αS was analyzed through MTT viability assays against the Raw 264.7 cell line. The results showed a specific cytotoxic activity of IL-33αS against this cell line, increasing its cytotoxicity as the incubation time and protein concentration increased (Figure 7), with an IC_50_ of 300 nM at 72 h. Moreover mIL-33 showed a small cytotoxic effect compared to the immunotoxin at the different times and concentrations assayed, presenting an IC_50_ at 72 h higher than 5 µM.

### 2.6. Immunological Characterization of the IL-33αS

**Figure 8:**
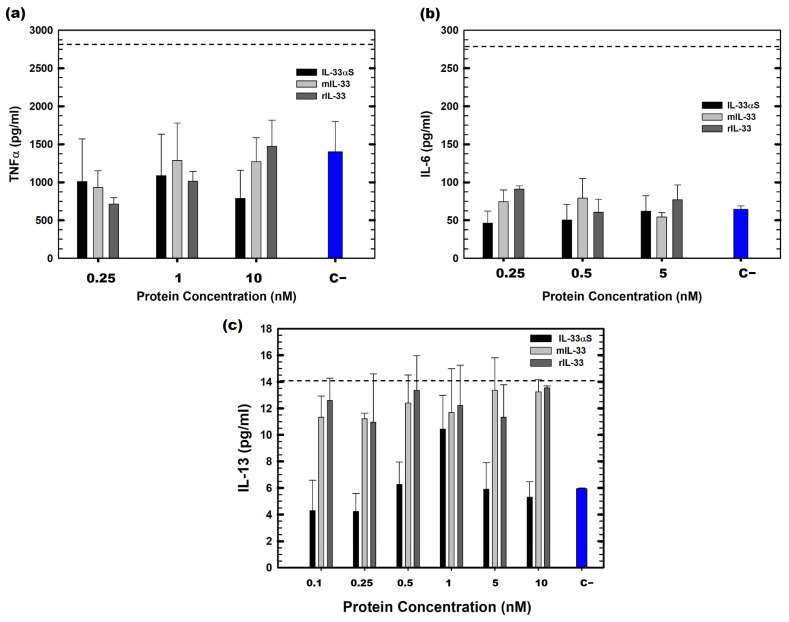
Cytokine secretion by Raw 264.7 after the stimulation with IL-33αS (black), mIL-33 (white), and rIL-33 (gray). IL-6 (**a**), TNF-α (**b**), and IL-13 (**c**) cytokines were analyzed through ELISA. The horizontal dotted line corresponds to the activation threshold representing the cytokine secretion caused by LPS (10 ng/mL). Measurements were analyzed and plotted (mean ± SD). For (**a**,**b**), quadruplicate tests of each condition were carried out, whereas duplicates were used for (**c**). Cytokine secretion of untreated conditions (C−) is represented in dark blue.

The immunological effect of IL-33αS on the Raw 264.7 cells, a murine macrophage-like cell line, was determined by analyzing the cytokine profile secreted by the cells when incubation with IL-33αS or mIL-33 took place. In addition, mature murine IL-33, commercially produced in *Escherichia coli*, rIL-33 (BioLegends, San Diego, CA, USA), was used as a control to demonstrate that mIL-33 produced in *Pichia pastoris* exhibited a similar immunological response as the commercial one. The cytokines that were selected for the study were TNF-α and IL-6, as an example of acute phase inflammation [26,27], and IL-13 as a typical Th2 inflammation cytokine [28]. Both IL-6 and TNF-α (Figure 8a,b), the cytokine secretion for all the proteins tested (IL-33αS, mIL-33, and rIL-33), was below the activation threshold, caused by the LPS, used as a positive control for unspecific immunologic activation. However, when IL-13, a typical Th2 cytokine secreted by IL33/ST2 signaling, was tested, both mIL-33 and rIL-33 showed a similar release of this cytokine compared to the LPS-treated condition. On the contrary, IL-33αS exhibited a much lower IL-13 secretion compared to both mIL-33 and rIL-33, and LPS (Figure 8c).

## 3. Discussion

Immunotherapy, based on immunotoxins, is a promising therapeutic strategy against a wide range of diseases, having been further developed against solid and hematological tumors [29,30]. However, very few immunotoxins have been developed against allergic conditions, with few exceptions, such as some designs against mite allergy [23] or aimed against IgE-positive cells [31]. The absence of specific antigens that are widespread in allergic responses but absent in physiological conditions constitutes the main obstacle for specific allergic immunotoxins [32], aside from issues derived from complex etiologies of allergy, individual patient variation, long-term therapy administration, and cost and accessibility of treatment. An ideal allergic immunotoxin should target an antigen overexpressed in allergic-involved immune cells compared to healthy tissue, and exhibit low immunogenicity, as it happens with ribotoxins [33].

In this sense, we have designed, produced in *Pichia pastoris*, and purified a new immunotoxin against allergic inflammation using the cleaved active form of the murine IL-33 that binds to the ST2 receptor, which is crucial in allergic inflammation, especially in type 2 inflammation. Unlike type 1 inflammation, which usually exhibits a rapid and transient inflammation, type 2 inflammation tends to progress slowly and remain persistent, causing atopic dermatitis and mainly respiratory diseases, such as asthma, pulmonary fibrosis, and allergic rhinitis [34].

In addition, we have produced the cleaved active form of the murine IL-33 for its use as a control, also in the yeast *P. pastoris* (also known as *Komagataella phaffii*). The use of *P. pastoris* as an expression system instead of *E. coli* has several advantages. First, the glycosylation pattern produced by the yeast is more similar to the human one, resulting in a decrease in the immunogenicity of the proteins [35], allowing repeated doses of therapeutic administration without the development of anti-drug antibodies (ADAs) by the patient, which would eventually reduce and limit its medical efficiency [36]. On the other hand, it allows a better formation of disulfide bonds in proteins, such as α-sarcin. In addition, it is considered a GRAS (Generally Recognized As Safe) organism [37]. Our group has extensive experience in the use of *P. pastoris* as an expression system to produce different antibodies fused to α-sarcin [14,21].

Both proteins have been successfully produced and purified through affinity chromatography, with final yields of 5 mg/L for IL-33αS and 15 mg/L for mIL-33. The significant increase in the production of mIL-33 compared to IL-33αS can be explained due to its smaller size and the absence of the toxic domain, which could interact with the ribosome of the yeast, leading to a slight decrease in viability and in its production [38]. The Western blot analysis showed that both proteins kept the identity of the target domain, as they reacted with the corresponding specific antibodies. The slight degradation observed in IL-33αS corresponds to degradation from the C-terminus of the protein, as it also appears in the Western blot using the anti-α-sarcin polyclonal antibody.

The structural characterization of IL-33αS, by means of the circular dichroism spectra, showed that it exhibited a majority of β-sheet secondary structure, which is consistent with the predominant secondary structure of the cytokine IL-33 [10,39] and the ribotoxin α-sarcin [40,41]. In addition, the size-exclusion chromatography showed that IL-33αS eluted in a major peak, at the expected size of 40 kDa, indicating that it consisted of a monomer at its native state. This was also reported for the mIL-33 alone, showing a main peak corresponding to 20 kDa.

The functional characterization of both IL-33αS and mIL-33 indicated that IL-33αS exhibited ribonucleolytic activity due to the presence of α-sarcin, releasing the characteristic 300–400 nucleotide rRNA fragment, called α-fragment, increasing the ribonucleolytic activity and the intensity of the band as the amount of protein was assayed, in a similar way to other immunotoxins already assayed in our group [33,42]. However, mIL-33 did not exhibit any ribonucleolytic activity, as expected. As for the target domain, both mIL-33 and IL-33αS were able to specifically bind to the ST2 receptor present on the Raw 264.7 cell line [43].

The combined action of both domains was assessed through viability assays, since once the target domain of IL-33αS binds to the ST2 receptor on the surface of the immune cells, it is then internalized through endocytosis, following the retrograde route via the Golgi apparatus and finally being translocated into the cytosol, where the toxic domain can exert its ribonucleolytic activity on the ribosomes, leading to cell death [44,45]. IL-33αS exerts its cytotoxic activity against the Raw 264.7 cell line, with an IC_50_ of 300 nM, in a similar cytotoxic range to other immunotoxins targeted against tumoral antigens [46]. Moreover, we have also characterized the cytotoxic effect of another immunotoxin based on Der p 1 allergen and α-sarcin ribotoxin (pro Der p 1-αS), as well as α-sarcin alone against Raw cells [23]. Table 1 shows the percentage of viability obtained for these constructs and the ones described in this work, after incubation for 72 h, with 1 mM protein. IL-33αS has a much higher cytotoxic efficacy than that observed with the other immunotoxin.

On the other hand, the lack of cytotoxicity observed after incubation with ribotoxin alone demonstrates that IL-33αS exhibits a highly specific cytotoxicity, targeted by the binding of IL-33 to the ST2 receptor. This specific cytotoxicity, with the adjustment of the dose of IL-33αS administered, could reduce or mitigate the high response of activated macrophages.

As for the immunologic characterization, we tested both the IL-33αS and mIL-33 produced in *P. pastoris*, and a commercially available recombinant IL-33 produced in *E. coli* (rIL-33), to determine if the IL-33 produced in the yeast triggered the same immunological response as the commercially available one. Neither of the proteins produced secretion of TNF-α or IL-6 above the activation threshold, which are typical pro-inflammatory cytokines [47]. However, regarding IL-13, which is a characteristic Th2 inflammation cytokine produced by Th2 lymphocytes, mast cells, macrophages, and ILC2 upon stimulation of IL-33, among others [34,48], we observed that both IL-33, produced in bacteria and in yeast, secreted levels of IL-13 in a similar way to the activation control by LPS. However, the administration of IL-33αS induced a much lower secretion of this cytokine.

**Figure 9:**
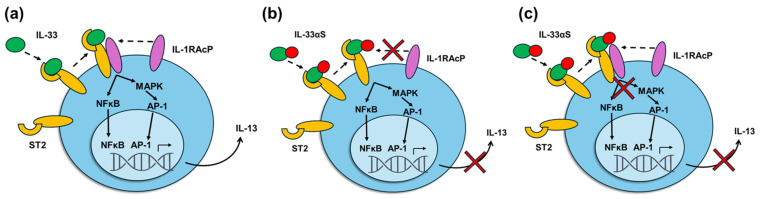
Schematic diagram showing the activation of immune cells upon stimulation by IL-33 or IL-33αS. When IL-33 (**a**) binds to the ST2 receptor, and it is activated with the presence of the coreceptor IL-1RAcP, the signaling pathways, including NFκB or MAPK, take place, and conclude with the secretion of Th2 cytokines. In the case of IL-33αS, two scenarios are proposed when bound to ST2: the presence of α-sarcin impairs the binding to the coreceptor (**b**) or prevents the activation of the ternary complex (**c**), avoiding in both cases the corresponding signaling pathways and the final secretion of Th2 cytokines.

We postulate that the presence of the α-sarcin at the C-terminal end of the protein affects the formation or activation of the IL-33/ST2/Il-1RAcP signaling ternary complex. This may be due either to the presence of steric impediments that prevent the recruitment of the IL-1RAcP coreceptor or prevention of the activation of the receptor (Figure 9). In the first case, the presence of the toxin would affect key residues at the ST2-IL-1RAcP interface. Thus, the toxic domain could act as a negative regulator of the IL-33/ST2 pathway, similar to the SIGIRR (single immunoglobulin IL-1R-related molecule) receptor, also known as IL-1R8 or TIR8, which interferes with the recruitment of the IL1RAcP coreceptor [49,50]. In the second case, key IL-33 residues involved in the activation of the signaling pathway would be affected. In both cases, a decrease in cytokine release levels would be observed. Although we do not have experimental data that allow us to confirm which of the two situations occurs, the results obtained previously on the structures obtained in the IL-33/ST2 and IL33/ST2/IL-1RAcP binding complexes [39,51,52] seem to indicate that the activation of the ternary complex would be affected rather than the formation of this complex.

In this regard, the importance of the amino-terminal end and residues of the β3 and β4 chains of IL-33 for the formation of electrostatic interactions at the binding interface of the D3 domains of ST2 and IL-1RAcP has been described [39,51] and thus is critical for binding affinity and stability. However, activation of ST2 signaling depends on interactions with the carboxyl-terminal end, the β1-terminus, the β2-β3 loop, and β12 [51,52].

**Figure 10:**
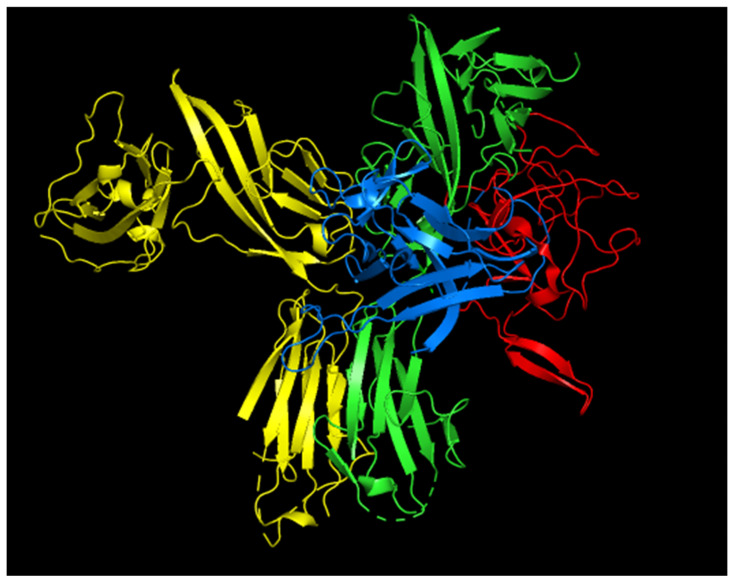
Structural model of the complex IL-33αS/ST2/Il-1RAcP created with PyMol. Color code: IL-33 (blue); α-sarcin (red); ST2 receptor (green); and IL-1RAcP (yellow).

Using the PyMol software v2.5.7. we have created a theoretical structural model of the IL-33αS/ST2/IL-1RAcP complex from the already published structures of the ternary complex, including at the C-terminal end of IL-33, the structure of α-sarcin (Figure 10). As observed in the model, α-sarcin does not appear to affect the interaction zones between ST2 and IL-R1ACP. However, it would significantly affect the IL-33 residues responsible for the structural fit for ST2 activation.

On the other hand, the results described for a fusion protein formed by IL-33 bound by its C-terminal end to the mannose-binding protein (MBP) showed that the formation of the ST2-IL-1R1AcP complex was not affected [39], although they did not perform signaling activation studies. Moreover, point mutants of IL-33 at different residues have been described, showing significant differences in binding or activation, respectively. Thus, IL-33D175A presents a significant decrease in the affinity and binding of the complex without affecting the signaling pathway, while IL-33L264A, close to the carboxyl-terminal end, does not produce alterations in the binding of the complex, but presents a very significant decrease in the activation of signaling [51]. The results we have obtained, together with the structural model developed, seem to suggest that the α-sarcin present in our construct modifies the structural arrangement of IL-33, preventing conformational rearrangement for ST2 activation.

Thus, the results obtained from the design, production and purification of this new immunotoxin IL-33αS support its potential effect as a therapeutic approach in allergic inflammation, both for its cytotoxic effect when the immune cells are in an inflammatory state, and for the decrease in Th2 cytokines secreted by these cells, suggesting that the use of immunotoxins could be an effective and promising therapeutic tool in allergic-derived pathologies.

Further investigations using allergic mouse models will be carried out to assess the in vivo relevance of this new immunotoxin. Future experiments will consist of studying the effect of both proteins (both mIL-33 and IL-33αS) on splenocytes from allergic and control mice, and studying the differences in immune cell populations (such as CD4+, CD8+, or helper T lymphocytes), and their cytokine secretion profile. In addition, we need to determine if the protective effect on the Th2 inflammation caused by IL-33αS is maintained at the organism level, where it could affect the proliferation and differentiation of the different lymphocytes [53,54], or the recruitment of immune cells, such as lung natural helper cells or eosinophils.

On the other hand, concerns may be raised about the potential adverse effects of IL-33αS signaling inhibition in other cell types. In this regard, it should be noted that although the ST2 receptor is expressed in endothelial and epithelial cells [55,56], the level of expression is lower than that observed in T helper 2 (Th2)-related immune cells such as Th2 cells, mast cells, basophils, and eosinophils [57]. Thus, it is expected that IL-33αS acts primarily on tissue-resident immune cells that constitutively express ST2 [58]. In this regard, the potential application of the cytokine IL-33 has already been described not only in therapeutic strategies for allergies, but also against cancer and other types of inflammatory diseases [59,60], suggesting the potential application of the whole immunotocin against allergy.

Nevertheless, this issue should be considered as the characterization of the potential therapeutic application of this construct is further investigated.

## 4. Materials and Methods

### 4.1. Plasmid Design

Both mIL-33 and IL-33αS cDNAs were provided by Integrated DNA Technologies, Inc, along with the desired restriction sites. For IL-33αS, a linker consisting of Gly2Arg was used to bind the mIL-33 domain and the α-sarcin domain. The cDNAs were then cloned into the pPICZαA plasmid (Invitrogen, Carlsbad, CA, USA) to finally obtain the plasmids pPICZαAmIL33 and pPICZαAIL33αS, and were later sequenced by the Genomic Unit at the University Complutense of Madrid, to confirm the sequences were correct. Both plasmids contained a zeocine resistance gene, an α-factor peptide signal at the N-terminus site, to allow the extracellular secretion, and a 6-histidine tag at the C-terminus site, to facilitate its purification.

### 4.2. Protein Production and Purification

Electrocompetent *Pichia pastoris* KM71H strain cells were electroporated with 10 µg of the linear plasmid (pPICZαAmIL33 or pPICZαAIL33αS) using a Bio-Rad Gene Pulser device (Bio-Rad, Hercules, CA, USA), according to the manufacturer’s instructions. Multiple clones were isolated against media with different concentrations of zeocine (100–750 µg/mL).

Production of the recombinant proteins was carried out as previously described [33], starting with 100 mL of pre-inoculum that was added to baffled flasks containing 2 L of BMGY media and incubated at 30 °C. After 24 h of culture, and with an O.D. > 5 A.U., the yeast cells were harvested and resuspended in 1 L of BMMY induction medium containing PMSF 1 mM and Methanol (0.5% *v*/*v*) at 15 °C. After 24 h, the extracellular medium was dialyzed several times against a final volume of 10 L of 50 mM sodium phosphate buffer containing 0.1 M NaCl, pH 7.5.

The purification of both proteins was carried out using an affinity chromatography with a Ni^2+^-NTA agarose column (GE Healthcare, Uppsala, Sweden). The dialyzed extracellular medium was loaded into the column using a peristaltic pump at a flow rate of 1 mL/min. After washing the column with phosphate buffer and with imidazole 20 mM in phosphate buffer, the proteins of interest were eluted using sodium phosphate buffer with imidazole 250 mM. The different fractions were analyzed through SDS-PAGE (15%) followed by Coomassie blue staining, or Western blotting using an anti-murine IL-33 monoclonal antibody (Fisher Scientific, Pittsburgh, PA, USA) or a rabbit polyclonal anti-α-sarcin antibody.

### 4.3. Structural Characterization

Absorbance measurements of the proteins were carried out using a UV-1800 spectrophotometer (Shimadzu, Shimadzu Europa GmbH, Duisburg, Germany). Far-UV circular dichroism (CD) analysis was obtained through a JASCO J-175 spectropolarimeter (Jasco Analitica, Madrid, Spain) at a scanning speed of 40 nm/min. Proteins were dissolved in phosphate buffer, NaCl 0.5 M, at a final of 0.15 mg/mL. At least six spectra were obtained and averaged to obtain the final concentration result. Size-exclusion chromatography was performed by FPLC in an AKTA purifier device (GE Healthcare, Lifescience, Marlborough, MA, USA) using a Superdex 200 Increase 10/300 column (Cytiva, Marlborough, MA, USA) at a flow rate of 1 mL/min.

### 4.4. Ribonucleolytic Activity Assay

The highly specific ribonucleolytic activity of α-sarcin, present as the toxic domain of IL-33αS, was assayed as previously described [61,62]. The ribonucleolytic release of the 400 nt rRNA fragment, known as the α-fragment, was used to analyze the ribonucleolytic activity of α-sarcin. Briefly, a rabbit cell-free reticulocyte lysate (Promega, Madison, WI, USA) (50 µL) was incubated with different amounts of the desired proteins. The ribonucleolytic reaction was stopped by the addition of 250 µL of Tris 50 mM, SDS 5% (*w*/*v*), pH 7.5 buffer, then the RNA was isolated with 300 µL of a phenol/chloroform/isoamyl alcohol solution (25:24:1) and precipitated by the addition of another 300 µL of isopropanol. After 24 h at −20 °C, the precipitated RNA pellet was resuspended in 10 µL of DEPC H_2_O. The presence of the characteristic α-fragment was visualized by electrophoresis in a 2% (*w*/*v*) agarose, 16% (*v*/*v*) paraformaldehyde gel, prestained with ethidium bromide. Images were finally obtained using a Bio-Rad Universal Hood II Transilluminator device (Bio-Rad, Hercules, CA, USA), and images were analyzed using the Quantity One software v4.6.9. (Bio-Rad, Hercules, CA, USA).

### 4.5. Cell Line Cultures

The Raw 264.7 cell line (TIB-71, from American Type Culture Collection)(Rockville, MD, USA) was used as a ST2^+^ murine macrophage cell line [24,25], whereas the Hela cell line (human cervix adenocarcinoma, CCL-2, from American Type Culture Collection) was used as a ST2^−^ fibroblast-like cell line. Both cell lines were cultured in DMEM medium, supplemented with 300 mg/mL of L-glutamine, 50 mg/mL of streptomycin, 50 µg/mL of penicillin, and 10% (*v*/*v*) fetal bovine serum. The cells were cultured at 37 °C in a humidified atmosphere (CO_2_/air, 1:19. *v*/*v*). Harvesting and propagation of both cell lines were performed every 3–4 days through mechanical scraping. The number of cells was calculated with a Neubauer chamber.

### 4.6. Flow Cytometry Assays

Prior to the flow cytometry assay, purified mIL-33 and IL-33αS were labeled with Alexa Fluor 488, using the Alexa Fluor 488 labeling kit (Fisher Scientific, Pittsburgh, PA, USA), obtaining mIL33-Alexa488 and IL33αS-Alexa488.

After subculture, the cells were distributed in aliquots of 3 × 10^5^ cells/mL and washed three times with 300 µL of 1% (*w*/*v*) BSA-PBS. Then the cells were incubated with different concentrations of the desired proteins conjugated with Alexa488 using gentle agitation for 1 h in the dark. Incubation with just an anti-Histag 488 was used as a control isotype. Then, the cells were harvested via gentle centrifugation (1200× *g* at 4 °C, 10 min), washed again three times with 1% (*w*/*v*) BSA-PBS, and fixed with 1% (*v*/*v*) paraformaldehyde. Flow cytometry acquisition was carried out using a FACScan device (Becton Dickinson, Franklin Lakes, NJ, USA) at the Centro de Apoyo a la Investigación of the Universidad Complutense de Madrid. The results were analyzed using the FlowJo software (FlowJo v10, Oregon, OR, USA).

### 4.7. MTT Viability Assay

The cytotoxic activity of the new immunotoxin, IL-33αS, or the recombinant cytokine mIL-33 against its target cell was measured by an MTT viability assay using a commercial kit (Roche, Mannheim, Germany). The Raw 264.7 cells (ST2^+^) were seeded at a density of 5 × 10^3^ cells/well and incubated at 37 °C for 24 h. The medium was removed, and different concentrations of IL-33αS or mIL-33 were added to the cells at a final volume of 200 µL of medium without serum. Wells were incubated for 24, 48, and 72 h. After the incubation, 20 µL of MTT (5 mg/mL) was added to each well for 3 h. Then, 100 µL of Solubilization Buffer was added to each well to dissolve the formazan crystals formed by the reduction in MTT carried out by the enzymatic activity of live cells. A total of 24 h after the solubilization, the results were obtained by measuring the optical density at 570 nm of each well and expressing it as a viability percentage. The medium-cultured cells were used as the 100% viability control, and triplicate samples of each condition were tested. IC_50_ value was established as the concentration of immunotoxin that led to a 50% viability decrease.

### 4.8. Immunological Analysis

The immunological characterization of both mIL-33 and IL-33αS was carried out by analyzing the cytokine profile secretion of the Raw 264.7 cells cultured with the different proteins. The cells were cultured at a 10^5^ cells/well density for 24 h in medium. After 24 h, different concentrations of proteins (mIL-33 and IL-33αS) were added to serum-free medium for 24 h. Recombinant commercial IL-33 (BioLegends) was also added as a physiological control. Also, LPS from *Eschericha coli* 10 ng/mL was added as a positive immunological control. Then, supernatants of the different conditions were collected for the cytokine analysis. TNFα, IL-6, and IL-13 were tested via ELISA, following the manufacturer’s instructions (Invitrogen, Camarillo, CA, USA). Quadruplicates or duplicates for each condition were tested. ANOVA with a post hoc analysis using the Student–Newman–Keuls test was used for statistical analysis within each test to compare the results obtained with the different doses administered. Differences between the experimental groups were considered statistically significant at *p* < 0.05.

### 4.9. Equipment and Settings

The gel images from Figure 2a,b, Figure 3a and Figure 5 were acquired and analyzed using the Gel Doc XR Imaging System and Quantity One 1-D analysis software v4.6.9 (BioRad, Hercules, CA, USA). The blot image from Figure 3b was acquired and analyzed using ChemiDoc-It (UVP) (Upland, CA, USA) and VisionWorks LS analysis software v7.0. If processing in brightness and contrast of gel and blot images was performed, it was applied to the entire image, including controls. No high-contrast gels or blots was displayed. When necessary, cropped gels were displayed to improve the clarity and conciseness of the presentation, being indicated in the figure.

SigmaPlot—Scientific Data Analysis and Graphing Software v11 (Systat Software Inc.) was used for graphing of experimental data in Figure 7 and Figure 8.

### 4.10. Structural Model of IL33/ST2(IL-1RAcP)

The theoretical structural model of the chimeric IL-33αS protein, and ST2 receptor complex and its interaction with IL-33, were obtained from the already-known NMR structure of α-sarcin (PDB: 1DE3; DOI: 10.1006/jmbi.2000.3813) and the X-ray diffraction structure of IL-33/ST2/IL-1RAcP ternary complex (PDB: 5vi4; DOI: 10.1016/j.immuni.2017.08.004). Using the PyMol Molecular Graphics System 3.0 (Schrödinger, LL.), the α-sarcin structure was fused to the IL-33 structure, creating a unique covalent bond between the IL-33 C-terminal and the α-sarcin N-terminal using the builder module of the software. Original structures were not modified by this bond, and the relative position between them was predicted, allowing the program to model the free angles of the introduced bond, delimited by the steric impends and protein surface charges. IL-33αS was aligned with the IL-33 structure of the IL-33/ST2 complex for steric impend visualization when the chimeric protein tries binding to the receptor.

## 5. Conclusions

The work described herein represents an innovative approach towards the treatment of allergy, based on a new immunotoxin (IL-33αS) targeted against ST2. This new immunotoxin was able to specifically bind to ST2^+^ cells, leading to cytotoxicity caused by α-sarcin as the toxic domain. In addition, the binding of IL-33αS to the ST2^+^ cells led to a decrease in the Th2 cytokine secretion, probably due to the presence of α-sarcin that prevents the full activation of the ST2-IL-1R1AcP complex, suggesting an important step towards the therapeutic approach against allergy.

## Figures and Tables

**Table 1:** Comparison of the cytotoxic efficacy of different constructs on Raw 264.7 cells. The values shown for α-sarcin and Derp1-αSarcin were obtained from [21].

Protein/Immunotoxin (1 µM)	Viability (%) on Raw 264.7 Cell Line
α-Sarcin	95
proDerp1-α-sarcin	60
mIL-33	85
IL-33αS	40

## Data Availability

All the experimental data generated or analyzed during this study are included in the article.

## Supplementary Materials

The following supporting information can be downloaded at https://www.mdpi.com/article/10.3390/ijms26199827/s1.
