# Supplementary material for: An Innovative Immunotoxin Design Against Allergy Based on the IL-33 Cytokine and the Ribotoxin α-Sarcin
Source: Int J Mol Sci. 2025 Oct 9;26(19):9827. doi: 10.3390/ijms26199827 (PMC12525494; doi:10.3390/ijms26199827)
Supplement: Supplementary file 1 [file ijms-26-09827-s001.zip › ijms-3880545-supplementary.pdf]

An Innovative Immunotoxin Design Against Allergy Based on the IL-33 Cytokine and the Ribotoxin α-sarcin

Narbona, J.

Lázaro-Gorines, R.

Gutiérrez-Carmona, A.

López-Rodríguez, J.C.

Villalba, M.

Lacadena, J.

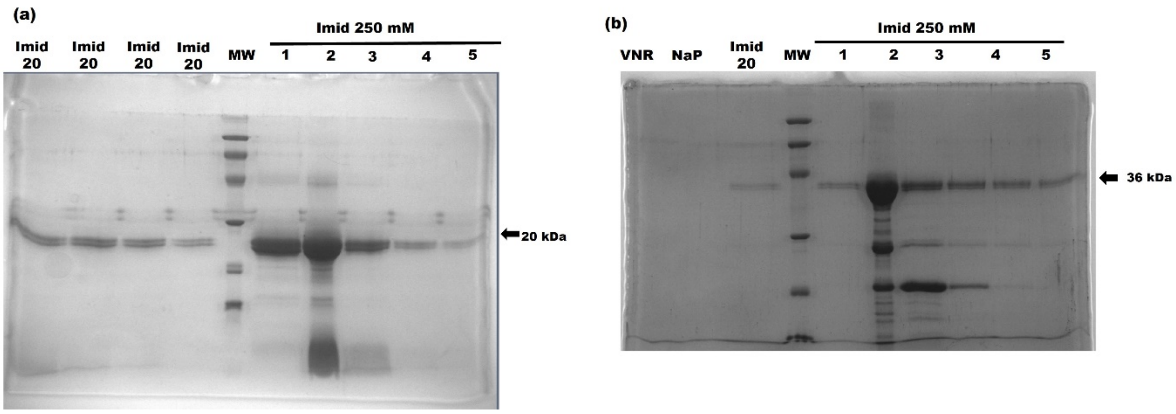
(a)
Imid 20
Imid 20
Imid 20
Imid 20
MW
Imid 250 mM
1
2
3
4
5
20 kDa
(b)
VNR
NaP
Imid 20
MW
Imid 250 mM
1
2
3
4
5
36 kDa

Figure S1

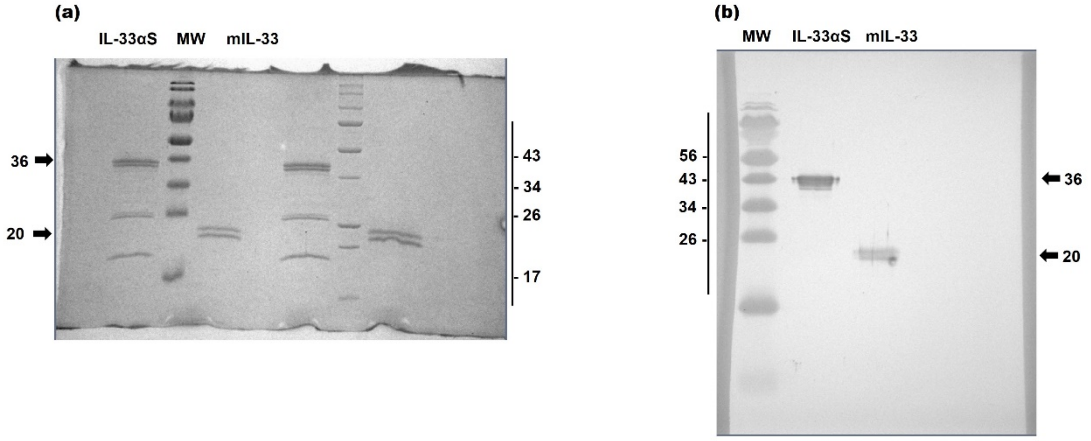
(a)
IL-33αS
MW
mIL-33
36
20
43
34
26
17
(b)
MW
IL-33αS
mIL-33
56
43
34
26
36
20

Figure S2

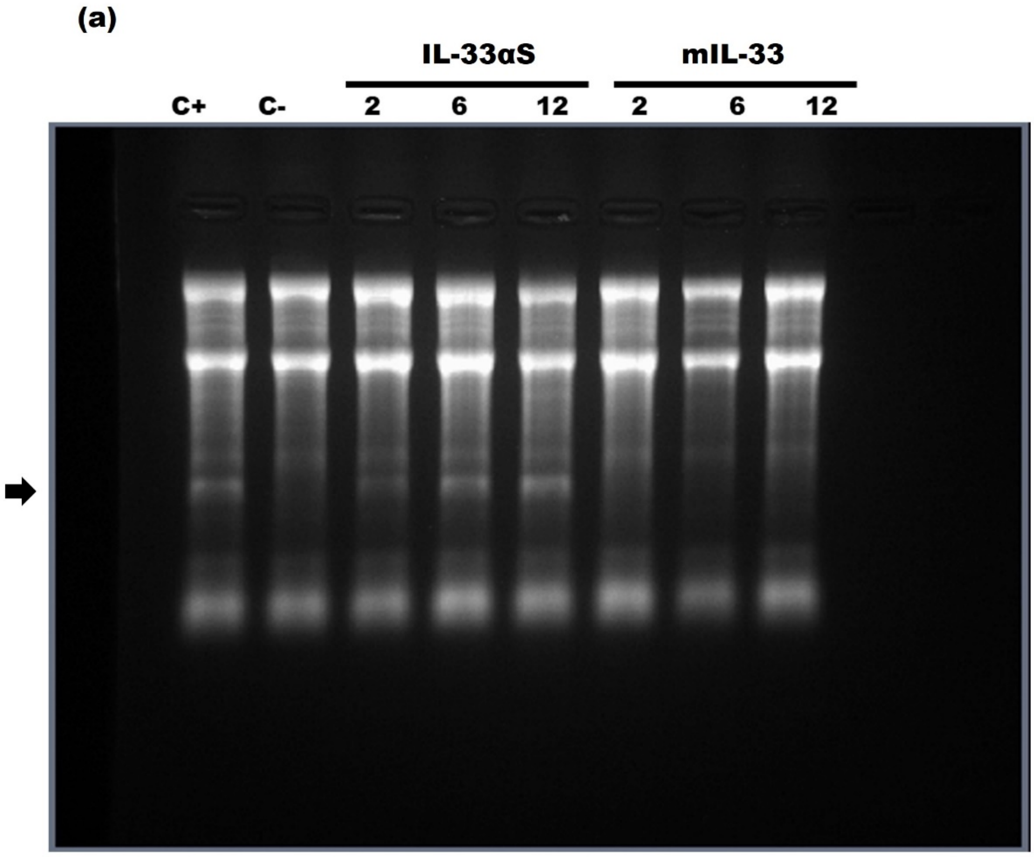

Figure S3

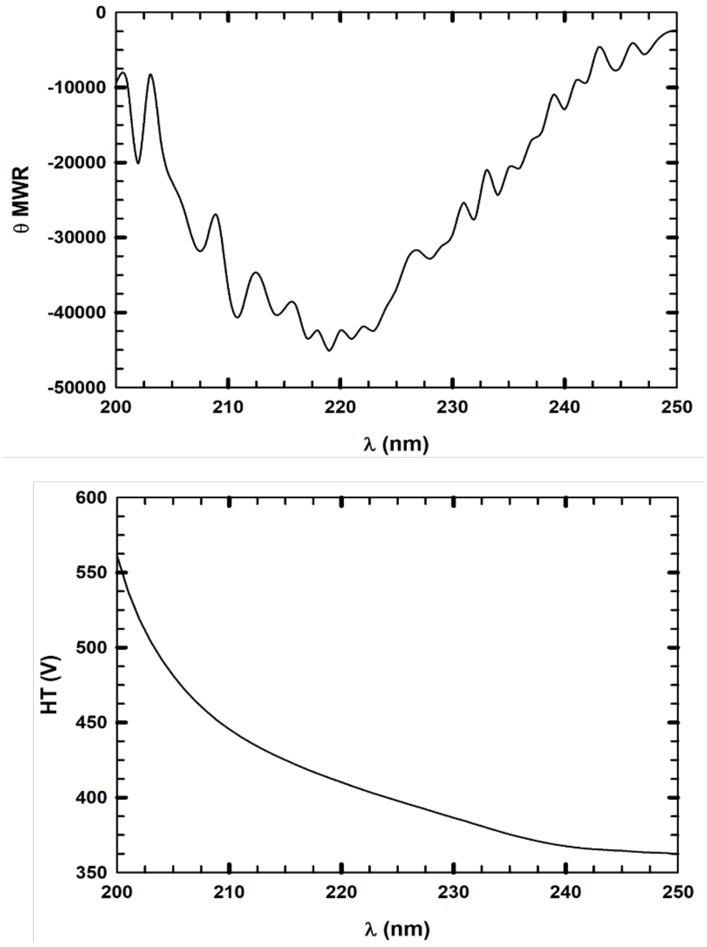

Figure S4

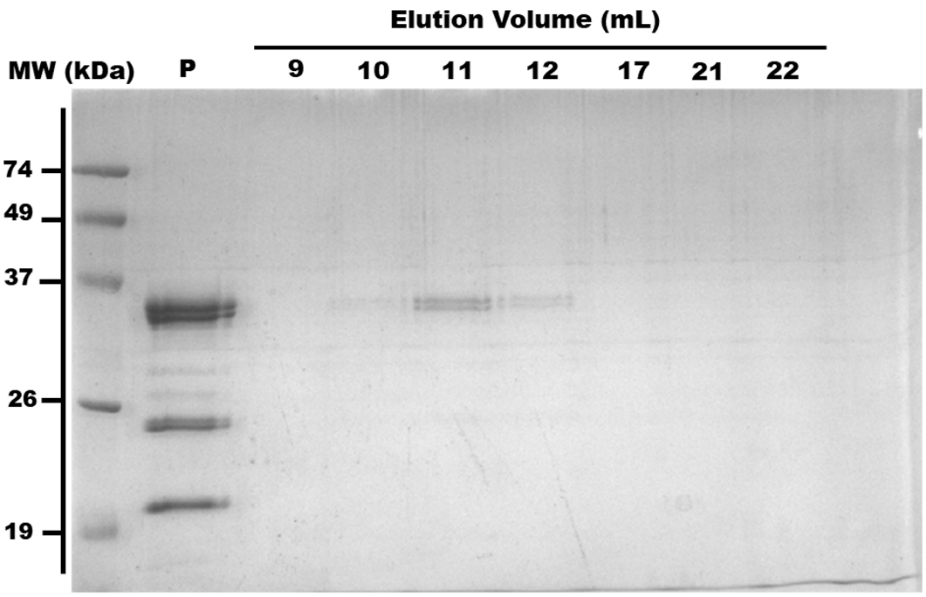

Figure S5

Supplementary Figures Legends

Figure-S1. SDS-PAGE of different fractions from affinity chromatography purification of mIL-33 and IL-33αS. Coomassie blue stained SDS-PAGE analysis of the purified fractions of the affinity chromatography of mIL-33 (a) and IL-33αS (b). Notes in gel correspond to the following: MW: molecular weight standard; NaP, washed fraction eluted with sodium phosphate buffer; Imid 20, washed fractions eluted with imidazole 20 mM sodium phosphate buffer; Imid 250 #1-5 and elution fractions with imidazole 250 mM sodium phosphate buffer. Molecular weight markers (MW) correspond, from top to bottom, to 74, 49, 37, 26, 19 and 11 kDa respectively. Original full-length gels from Figure 2 are presented**.**

Figure-S2. SDS-PAGE and Western Blot analysis of both purified proteins. a) Coomassie blue stained SDS PAGE analysis of the final pool at the end of the purification process of mIL-33 and IL-33αS. Molecular weight markers (MW) values appear at the right. b) Western Blot analysis of both the purified mIL-33 and IL-33αS, using an anti-IL33 monoclonal antibody. All molecular weights are shown in kDa. Original full-length gel and blot from Figure 3 are presented Images corresponding to gel and blot were acquired and analyzed using the Gel Doc XR Imaging System and Quantity One 1-D analysis software (BioRad) or ChemiDoc-It (UVP) and VisionWorks LS, respectively.

Figure-S3. **Functional characterization of the ribonucleolytic activity.** (a) The agarose gel represents the ribonucleolytic activity of α-sarcin in IL-33αS and mIL-33. The arrow indicates the presence of the α-fragment. In both cases, 2, 6 and 12 pmoles of protein were tested. C+ represents 2 pmoles of fungal wild-type α-sarcin, and in C- the protein sample was replaced by buffer. 2, 6 and 12 pmoles of IL-33αS and mIL-33 were tested. Original full-length gel from Figure 5a is presented. Gel image was acquired and analyzed using the Gel Doc XR Imaging System and Quantity One 1-D analysis software (BioRad).

Figure S4. Structural characterization of IL-33αS. (a) Far-UV circular dichroism (CD) spectra of IL-33αS, at a final concentration of 0.2 mg/ml in 50 mM sodium phosphate, 0.1M NaCl buffer, pH 7. θ_{MRW} represents the mean residue weight ellipticity as degree x cm^2 x $dmol^{-1}$. (b) HT signal obtained during Far-UV circular dichroism spectra measurements.

Figure-S5. SDS-PAGE of different fractions from IL-33αS SEC analysis. Coomassie blue stained SDS-PAGE analysis of selected fractions of FPLC SEC analysis. Lines shown correspond to: MW, molecular weight standard; P, IL-33αS pool obtained from the affinity chromatograpy step purification; Elution volume fractions (numbers correspond to elution volumes shown in figure 4b).
